# Mindfulness and foreign language achievement: a meta-analytic study on interventions and correlations

**DOI:** 10.3389/fpsyg.2025.1479462

**Published:** 2025-05-14

**Authors:** Muzaffer Pınar Babanoğlu, Erkan Hasan Atalmış

**Affiliations:** ^1^Department of English Language Teaching, Faculty of Education, Mersin University, Mersin, Türkiye; ^2^Department of Sports Sciences, Faculty of Education, Manisa Celal Bayar University, Manisa, Türkiye

**Keywords:** mindfulness in foreign language learning, mindfulness in education, foreign language learning and teaching, mindful language learning, mindfulness and second language learning

## Abstract

**Background:**

Mindfulness has recently gained attention for its potential to improve learning and teaching in foreign language education due to its ability to boost awareness and promote cognitive and emotional processes during language learning. To date, the significance of mindfulness has been investigated either through experimental studies with mindfulness interventions or correlational studies based on the connection between mindfulness scores and language learning. This article attempts to explore the overall effect sizes of (1) the impact of mindfulness interventions on foreign language achievement and (2) the relationship between mindfulness scale scores and language achievement through a meta-analytic review of the research perspective.

**Method:**

The meta-analysis includes experimental studies examining the effects of mindfulness interventions on foreign language performance and correlational studies examining the association between mindfulness scores and various aspects of language proficiency. From 10 countries, a total of 14 studies with 1039 participants for interventions and 9 studies with 2232 participants for correlational studies were tested through statistical meta-analysis procedures.

**Results:**

The findings showed that the mean effect sizes were significant (Hedges’ *g* = 0.67 for intervention studies, *r* between mindfulness scores and academic achievement = 0.22), demonstrating the efficiency of mindfulness. No significance was found in the publication bias assessment and the moderator analysis on regional effect.

**Systematic review registration:**

The OSF link of the study: https://osf.io/2gxrq.

## 1 Introduction

The 21st century has brought huge technological developments and advancements that significantly influence our lives. From communication to health and business to education, almost all components of our lives are now closely related to computers, the Internet, Artificial Intelligence, and other tech-based entities to make tasks faster and life easier. Despite this inevitable shift toward efficiency in work practices through technology, humanity continues to value traditional spiritual teachings such as mindfulness, which emphasize human awareness and wellbeing. In education, mindfulness fosters focus and emotional resilience in both learners and teachers and helps them both to engage more deeply with one another. As technology transforms education, mindfulness remains vital in nurturing cognitive and emotional development. It helps learners and educators navigate modern educational practices and provide innovative approaches to combining advanced technology with the educational processes that already exist.

Originally proposed by Langer (1989), the concept of mindfulness has become a topic of interest in many disciplines as it suggests techniques inspired by spiritual exercises from Eastern cultures. These often concentrate on using consciousness and awareness in the present moment to control emotions and overcome challenges in life by boosting resilience, strengthening the force of resilience, and supporting general wellbeing. The phenomenon was widely accepted by scholars from more traditionally Western ideologies in fields such as medicine and clinical psychology (Wells, 2002, 2005). Subsequently, due to its potential to improve learning, it has been recognized in other disciplines, such as in various fields of education, as it serves as a mode of perceiving mental components—including perceptions, sensations, cognitions, and affects (Walach et al., 2006). Thus, the importance of affective factors in learning and teaching is widely recognized since emotions are genetically connected to and affect cognitive skills such as attention, memory, decision-making, problem-solving, and critical thinking (Osika et al., 2022). Ergas and Hadar (2019) claim that there is proof of the effectiveness of mindfulness practices on various aspects of students’ and teachers’ lives, yet a clear conceptualization is still needed to uncover the associations between mindfulness and education. Mindfulness also contributes to foreign language education since it addresses some necessary aspects, such as affective factors, including motivation, anxiety, and self-confidence, and cognitive factors, including memory, recognition, and cognitive depth. These are all significant for language learning achievement (Onwuegbuzie et al., 2000). This study examines mindfulness in relation to foreign language achievement by synthesizing experimental and correlational studies through a meta-analysis. Specifically, the study aims to determine the overall effect sizes (ESs) of (1) mindfulness interventions on foreign language achievement and (2) the relationship between mindfulness and language achievement in correlational research.

## 2 Literature review

### 2.1 Mindfulness

Simply defined by Langer (1989) as the act of noticing new things through attention and awareness, the concept of mindfulness has been enhanced to include the purposeful, non-judgmental acceptance of the present moment (Kabat-Zinn, 1994) by a set of practices based on breath control and meditation techniques. Being aware of the actual moment by purposefully paying attention also requires accepting the self and things the way they are and enables the inner mechanisms that control reactions or responses appropriately with the help of breathing as an anchor to bring thoughts into the present moment (Wells, 2002). This process creates a positive effect that balances affective and cognitive abilities, resulting in a chain reaction where one calmly pays attention, acknowledges, and accepts the present moment without judging, then experiences fruitful emotional and cognitive outcomes. Mortimore (2017) points out that “Mindfulness aims to teach us how to enter a more nourishing frame of mind by reducing distractive and ruminative thoughts” (p. 19). Plentiful research over 25 years has revealed that mindfulness has overall benefits in developing competence, memory, creativity, positive affect, health, and longevity while decreasing stress and accidents (Langer, 2000). Particularly, studies have also shown that mindfulness has positive correlations with and a promoting effect on decision-making abilities (Maymin and Langer, 2021) and critical thinking (Noone et al., 2016); attention, memory, and executive function for academic performance (Liu et al., 2021); and the social skills and academic outcomes of adolescents with learning disabilities (Beauchemin et al., 2008).

### 2.2 The link between mindfulness and education

In education, learning is accepted as a lifelong process and comprises three domains formulated in Bloom’s Taxonomy (1956): cognitive domain (knowledge), which captures intellectual abilities such as recall and recognition; psychomotor domain (skills), which includes physical coordination and other motor skills; and affective domain (attitudes), which is based on feelings, values, and motivation (Hoque, 2016). The mindfulness approach, which originates in Buddhist spiritual doctrine and was transformed into a psychological coping strategy by Western scholars, serves pedagogy since it is positively associated with cognitive and affective abilities (Hayes et al., 2004; Karunananda et al., 2016; Schutt and Felver, 2020) to promote learning. According to Hyland (2011), mindfulness frees the mind from delusions and errors to enable genuine learning and cultivates awareness, which is a prerequisite for meaningful learning and teaching. The potential of mindfulness in educational contexts and student success was pointed out by Leland, “Mindfulness education appears to have a positive impact on academic performance by helping students – even those with learning disabilities – focus, be more organized, plan ahead, perform better on exams, and think critically” (2015, p. 23). Through contemplative exercises based on mindfulness, learners reconstruct meaning and take advantage of the learning process (Xue, 2023).

The application of mindfulness in education is mainly based on mindfulness-based interventions originally practiced in clinical psychology and psychiatry to cure mental health problems, such as stress, depression, and anxiety (Creswell, 2017; Bhattacharya and Hofman, 2023). Mindfulness interventions include specific techniques such as meditating with attention to breathing to connect with sensations or feelings while standing, sitting, lying down, or moving (Meiklejohn et al., 2012). In educational contexts, interventions have been carried out in public schools and universities for early-childhood children, adolescents, and young adults to cultivate learning and teaching by reducing stress and anxiety levels, academic performances, socio-emotional development, and social relationships of students (Hyland, 2013; Roeser, 2014; Indriaswuri et al., 2023; Smith, 2023; Erten and Güneş, 2024).

Studies have shown growing evidence of the effectiveness of mindfulness in academic achievement and student success (Caballero et al., 2019; Schutt and Felver, 2020; Dikmen, 2022; Indriaswuri et al., 2023; Mcbride and Greeson, 2023), yet some scholars have been cautious about mindfulness-based applications in education and argue their limitations in providing solutions for pedagogical issues (Sellman and Buttarazzi, 2020; Lee et al., 2024). Another issue of mindfulness is underlined by Ergas and Hadar (2019), who claim that mindfulness practice and mindfulness in education are two distinct spheres and should be handled separately; they further suggest a binary and paradoxical conceptualization of the role of mindfulness “as” education and “in” education.

All in all, the mindfulness approach is pertinent for education since the two share some common perspectives toward learning, as stated by Hyland (2011), who underlines the link between mindfulness and education: “both spheres involve the attention to and modification of consciousness and modes of thinking and both aim at a form of enlightened awareness which pays due attention to values and feelings” (p. 13). Hence, mindfulness practices may contribute to the academic and life skills of students (Hammad Al-Rashidi and Aberash, 2024), such as learning to learn, reflecting on their own learning, and making good life decisions to achieve overall academic and life goals. Furthermore, mindfulness practice as an everyday life doctrine should be integrated into the curricula of all educational levels to support students’ overall success.

### 2.3 The context of foreign language education

Learning a foreign language mainly depends on various complex theoretical and practical processes. The dynamic nature of foreign language learning requires learning target language knowledge with receptive and productive skills through gradual practice integrated with cognitive (attention and memory), meta-cognitive (learning strategies), and individual, psychological, or affective factors (motivation, self-esteem, and attitude toward the target language) to master it thoroughly. According to Onwuegbuzie et al. (2000), the factors directly related to foreign language achievement are cognitive (like aptitude), affective (anxiety and others), personality (locus of control), and demographics (age, gender, and previous foreign language experience). The conceptualization of mindfulness has significant relevance in foreign language education, as it encompasses different but complementary approaches that influence learning outcomes. Langer (1989) interpretation of mindfulness, which emphasizes cognitive flexibility and active engagement in noticing new linguistic patterns, promotes adaptability, creativity, and deeper cognitive processing, which are all key factors in language acquisition and proficiency development (Moafian et al., 2019). According to Kabat-Zinn’s (1994) mindfulness framework, it is rooted in non-judgmental awareness and meditative practices and, therefore, plays a crucial role in managing language anxiety, enhancing emotional regulation, and promoting a more conducive, affective environment for language learning. Recognizing these different perspectives highlights the multidimensional impact of mindfulness on language learning, shaping both the cognitive and emotional dimensions of learner success. Additionally, mindfulness meditation has been linked to improvements in working memory capacity as well. Research suggests that mindfulness training can improve the regulation of attention, leading to better encoding and retrieval of information (Skelly and Estrada-Chichon, 2021; Kang, 2024). This is particularly beneficial in language learning, where retaining new vocabulary and grammatical structures is essential to achieving success. By promoting a heightened state of awareness, mindfulness practices enable learners to process and remember language information more effectively.

Affective factors play a vital role in foreign and second language learning as they interact with cognitive and meta-cognitive components (Arnold, 2021). These factors include emotions, moods, attitudes, and characteristics such as anxiety, motivation, self-esteem, and personality, all of which influence learning outcomes (Hurd, 2008; Bao and Liu, 2021). Krashen’s (1982) “affective filter hypothesis,” which builds on the work by Dulay and Burt (1977) in the 1970s, remains a cornerstone of second language acquisition theory (Luo, 2024). Arnold (2021) emphasizes that affective factors are central in the language classroom, as students’ confidence has a direct impact on their learning. Awareness of these factors enables teachers to implement strategies that lower affective filters, thereby promoting more effective language acquisition. To achieve this goal, some recent approaches, such as positive psychology in foreign language learning and teaching, which was introduced by MacIntyre and Gregersen (2012), have provided opportunities to create paths that address affective factors and positively exploit them for the sake of language learners. The tenets and contributions of positive psychology variables in language learning and teaching have been explained and conceptualized by Wang et al. (2021) as academic engagement, emotion regulation, enjoyment, grit, resilience, and wellbeing. It is accepted that mindfulness is pertinent, contributes to positive psychology variables and interventions that provide positive outcomes (Allen et al., 2021), and has positive correlations with overall academic achievement (Alomari, 2023; Indriaswuri et al., 2023). Studies such as those by Charoensukmongkol (2019), and Morgan and Katz (2021) show that mindfulness practices, including meditation and focused breathing, help learners manage stress and anxiety in foreign language classes, leading to increased confidence and willingness to communicate. These effects contribute to a more positive and effective language learning experience. Additionally, mindfulness can have a positive impact on the motivation of language learners by promoting a present-focused and non-judgmental attitude. This attitude helps learners to engage more deeply with the material, reduces the fear of making mistakes, and increases their willingness to participate. As learners become more attuned to their learning experiences without self-criticism, their intrinsic motivation to practice and improve their language skills is enhanced (Ghanizadeh et al., 2019; Ulivia et al., 2022).

### 2.4 Significance and purpose of the study

The objective of this study is to examine whether mindfulness interventions and degrees of mindfulness capacities, measured by scales, are associated with foreign language education. To the author’s knowledge, there is a gap in the research field concerning the meta-analytic approach for experimental research undertaking mindfulness intervention and correlational studies based on the relationship between mindfulness and the different aspects of foreign language achievement. To gain a clearer understanding of the topic beyond past research, this study mainly relies on statistical analyses of total ESs that would contribute to the field. Thus, it aimed to gather the related intervention and correlational studies that yielded statistical results and carry out a meta-analysis of these results to obtain a total ES of mindfulness interventions and mindfulness scores associated with foreign language learning contexts.

To achieve this goal, the following research questions (R.Q.) are posed:

R.Q. 1. What is the effectiveness of mindfulness in achievement in foreign language education?

R.Q. 2. Does the effectiveness of mindfulness in achievement in foreign language education depend on region?

R.Q. 3. What is the relationship between mindfulness and foreign language education?

R.Q. 4. Does the relationship between mindfulness and foreign language education depend on region?

## 3 Method

This study was designed as a meta-analysis. Meta-analysis is a quantitative research method that systematically combines the statistical results of related studies on a subject in the literature (Borenstein et al., 2009; Cooper et al., 2009). As mentioned by Yıldırım and Şen (2019), although primary research conducted within a specific subject may reveal similarities or differences in findings, the value of a research synthesis study lies in its ability to integrate these results. In this study, the meta-analysis method was used to bring together the results of the intervention and correlational studies that examined whether mindfulness interventions by experimental research and individual mindfulness scores measured by scales in correlational research are associated with foreign language achievement by applying Moher et al.’s (2009) PRISMA guidelines and American Psychological Association (2010) guidelines. There is no technical obstacle to combining different study designs in the same analysis since the ES matters from a statistical perspective (Borenstein et al., 2009). The study chose a meta-analysis due to limitations in previous single-study samples, many of which had small or disparate samples from specific contexts, potentially limiting generalizability and understanding. Some meta-analytic studies (Zenner et al., 2014; Verhaeghen, 2023) have addressed the challenges posed by small samples in the field of education and mindfulness interventions. Meta-analysis combines statistical results from multiple studies, increasing power and providing a clearer, more accurate effect size. It also reduces the risk of biases or inconsistencies that may arise from small or heterogeneous samples.

### 3.1 Search, review, and retrieval of studies

The relevant studies were searched within electronic databases, namely, Google Scholar, ERIC, Clarivate Web of Science, PubMed, PsychINFO, Council of Higher Education Theses Center, ProQuest, Scopus, Springer Link, and Science Direct. A keyword search was done using the terms “mindfulness in foreign language education,” “mindfulness in EFL learning,” “mindfulness in foreign language learning and teaching,” “mindfulness+foreign language education,” and “mindfulness interventions in foreign language learning and teaching.” As initial review criteria, open-access research articles, unpublished theses, and refereed full-text conference papers released between 2015 and 2024 were reviewed through hand search. Studies on ‘mindfulness in foreign language education’ have proliferated in recent years, reflecting a growing interest in its impact on language learning (Zeilhofer, 2020). The selection of studies was guided by their emergence over time, with a focus on research published between 2015 and 2024 to capture recent advances and contemporary perspectives in education and psychology. After identification by keyword relevance, the obtained studies were scrutinized according to the inclusion criteria.

### 3.2 Rationale for the inclusion and exclusion criteria

To identify and select relevant studies eligible for the meta-analysis, the following inclusion and exclusion strategies were employed during the investigation:

1. The study was established on experimental and correlational designs. The experimental studies tested mindfulness interventions in foreign language contexts for various achievement aspects as dependent variables through control-experimental groups, while the correlational studies examined the relationship between mindfulness and achievement without intervening in the process.
2. Studies focused on participants’ learning of various foreign languages (English, German, and Spanish) other than their mother tongues.
3. Studies that yielded sufficient statistical information for calculating ES [participant numbers (*n*) and correlation coefficient (*r*) for correlational studies and participant numbers (*n*), means (*M*), and standard deviations (*SD*) for intervention studies].

Considering the relevance of the inclusion criteria, studies on mindfulness in foreign language education that were based on qualitative analyses, systematic reviews, and conceptual analyses were excluded from the contents of the meta-analysis. Qualitative studies were excluded from the meta-analysis because they lacked the quantitative data necessary to calculate effect sizes, which would compromise methodological rigor. While this decision may limit the exploration of nuanced perspectives, it ensures the validity and reliability of the quantitative synthesis. Additionally, studies that were not open access and lacked sufficient statistical measurement information (e.g., *n*, *r*, *SD*, or *M* values) were also not eligible for the meta-analysis.

**Figure 1:**
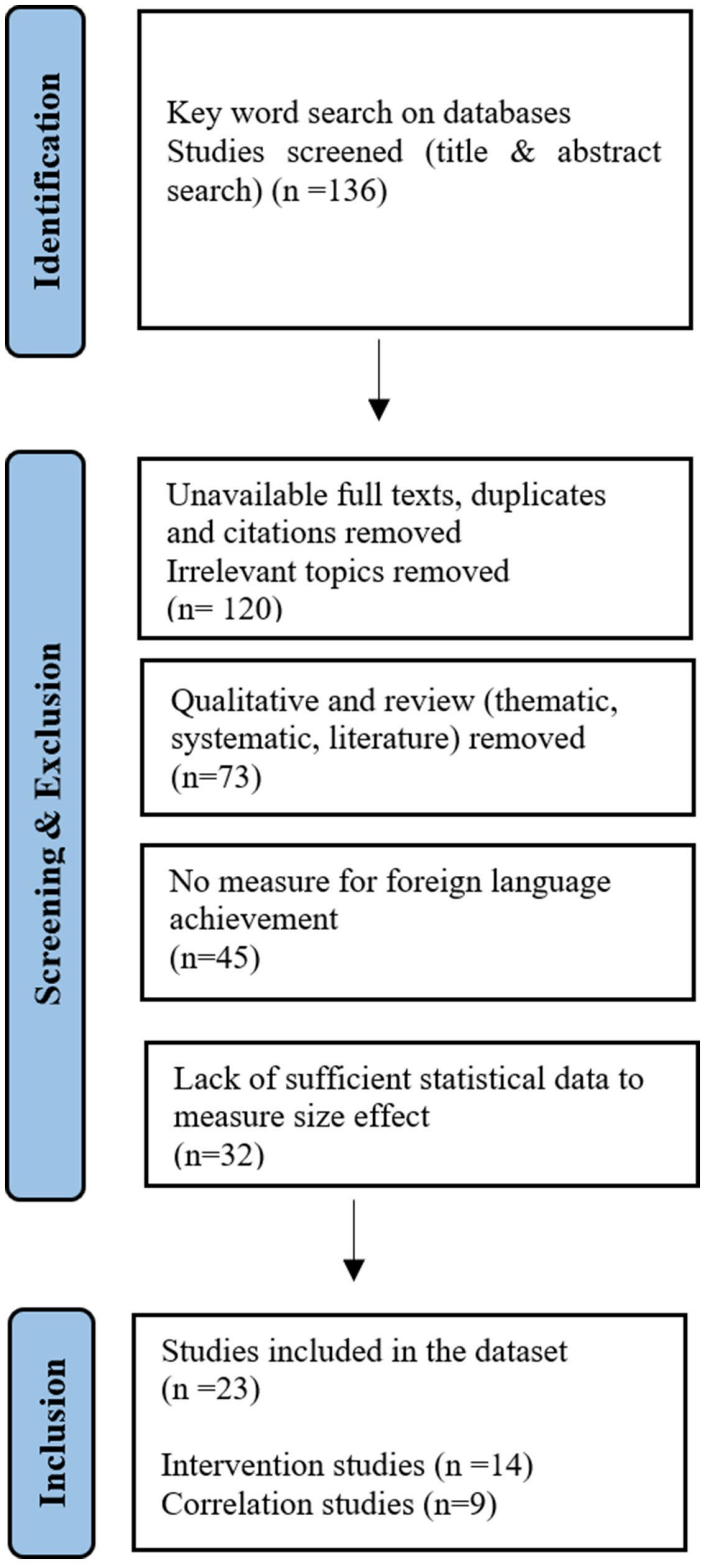
Steps in the data collection process. In Figure 1, the data collection process of the review phase is displayed.

The steps involved in the overall search and identification of eligible studies are displayed in Figure 1.

### 3.3 Study characteristics

After the initial identification process, a breakdown of main characteristics of relevant studies was done to see the overall picture. 23 studies from 10 countries with a total of 3271 participants were included in the data set. Selected studies focused on mindfulness through interventions or correlational relationships to explore possible effects on different aspects of language performance, ranging from general success to vocabulary recall or reading comprehension. Table 1 illustrates the characteristics of the studies selected for the meta-analysis.

### 3.4 Regional diversity

In the study, the possibility of regional effect was also measured by a moderator analysis to see whether mindfulness was applicable in different geographical regions and make the results more generalizable and heterogeneous as employed in other meta-analysis studies (Göçen and Şen, 2021; Chien, 2023). The mindfulness approach was derived from Eastern cultures long before it was transformed and began to be used in Western cultures lately. Most of the studies in the dataset were carried out in Eastern countries, which tells us that mindfulness is a recognized approach and regarded as a research interest in foreign language education as a part of the culture in these countries. Since the countries of selected articles examined mindfulness in different cultural and educational backgrounds and contexts, geography would be a distinctive variable. Since country scores in meta-analytic data are identified as a moderator variable (Hansen et al., 2022), to prevent a potential geographic selection bias for Eastern countries only, regional diversity in the study was considered as a variable, and studies from 10 countries were divided into two regional categories as Middle Eastern and Far Eastern countries in the moderator analysis.

### 3.5 Coding procedures

A coding form was developed for studies eligible for the meta-analysis to facilitate data analysis. Given the scope of the research, the authors’ names, publication year, regions (Middle Eastern and Far Eastern countries), sample size (*n*), and calculated values (*M*, *SD*, and *n*) were coded as variables into coding sheets created by two independent raters. An agreement index was calculated between the codes to calculate intercoder reliability, which was to be 0.90. The analysis was conducted after disagreements between the raters were discussed and resolved.

### 3.6 Effect size calculation

Two methods were applied to calculate the ES index for the meta-analysis in this study: SMD for the experimental (intervention) design studies and Pearson product–moment correlation (*r*) for the correlational design studies. The common way to calculate SMD is by Cohen’s *d*, computed as a combination of *n*, *M*, and *SD* for control and experimental groups, as shown below:


$$Cohen^{\prime }s\;d=\frac{M_{1}-M_{2}}{SD_{pooled}}$$

where $M_{1}$ , $M_{2}$ , and $SD_{pooled}$ represent the mean for group 1, mean for group 2, and the pooled standard deviation, respectively. The pooled standard deviation was computed as follows:


$$SD_{pooled}=\sqrt{\frac{\left(n_{1}-1\right)SD_{1}^{2}-\left(n_{2}-1\right)SD_{2}^{2}}{\left(n_{1}-1\right)+\left(n_{2}-1\right)}}$$

where $n_{1}$ and $SD_{1}$ represent the sample size and standard deviation for group 1 while and $n_{2}$ and $SD_{2}$ represent the sample size and standard deviation for group 2.

When the sample size is smaller than 20, Cohen’s *d* values are known to be biased. In this case, Hedges’ *g* value, an unbiased version of Cohen’s *d*, was applied as follows (Hedges, 1981):


$$Hedges^{\prime }g=d\times \left[1-\frac{3}{4\left(n_{1}+n_{2}\right)-9}\right]$$

where $d,n_{1}$ , and $n_{2}$ represent the Cohen’s *d* value, sample size for group 1, and sample size for group 2, respectively.

A positive Hedges’ *g* value suggests that the experimental group could have better achievement scores than the control group for the experimental design studies.

Moreover, Fisher’s *z* scale was applied for the Pearson correlation coefficient (*r*) to calculate the ES for each study as follows:


$$Fisher^{\prime }s\;z=\frac{1}{2}\log\left(\frac{1+r}{1-r}\right)$$

After all analyses were conducted by applying Fisher’s *z* transformation (i.e., ES), the results of the meta-analysis were converted back to the Pearson correlation coefficient as follows:


$$r=\frac{e^{2z}-1}{e^{2z}+1}$$

where *z* represents the Fisher’s *z* transformation value.

### 3.7 Statistical analysis

There are two methods commonly used in a meta-analysis: the fixed-effect model and the random-effects model (Hedges, 1992; Borenstein et al., 2009). In a fixed-effect model, it is assumed that each study in the meta-analysis belongs to a homogeneous population and the same ES parameter would be estimated, which is not exactly a realistic approach in social studies. Therefore, due to the assumption of heterogeneity, the random-effects model is preferred over the fixed-effect model (Lipsey and Wilson, 2001). Moreover, compared to the fixed-effect model, the random-effects model allows the results to be more generalizable.

The heterogeneity between studies is investigated by Q-statistics based on chi-square distribution, while the degree of heterogeneity was quantified by the *I*^2^ statistic (Higgins et al., 2003). Since *I*^2^ indicates the percentage of total variability in a set of ESs due to true heterogeneity (Huedo-Medina et al., 2006), it changes between 0% (no heterogeneity) and 100% (high-level heterogeneity). This value is computed as follows:


$$I^{2}=\frac{Q-k-1}{Q}\times 100$$

where *k* is the number of ESs.

In this study, the random-effects model approach was preferred to take assumptions of heterogeneity into consideration. After the model type (fixed-effect vs. random-effects) was decided, subgroup analyses were applied in cases of heterogeneity. Two subgroup analysis methods can be used in meta-analysis depending on moderator type. The ANOVA approach is proposed for the categorical moderators, while the meta-regression approach is recommended for the continuous moderators. In this study, the ANOVA approach was utilized to investigate whether mindfulness interventions and mindfulness scores are associated with foreign language learning based on region (Middle Eastern vs. Far Eastern countries), which is a categorical moderator.

### 3.8 Publication bias

Publication bias is a potential and common problem in meta-analyses that occurs when only published studies about a research topic are included. It is known that studies with significant and interesting results are more likely to be published than those without such characteristics (Sutton et al., 2000; Thornton and Lee, 2000). This means that a meta-analysis does not include unpublished studies, which may make the results invalid. Different methods are recommended to investigate the possibility of publication bias in meta-analyses, such as the funnel plot, Egger’s test, rank correlation test, and fail-safe N method. A funnel plot indicates the relationship between standard error and ESs. Publication bias is expected when the relationship is not symmetric/funnel-shaped (Light and Pillemer, 1984). The limitation of a funnel plot is that whether the shape is symmetric is informally defined and visually tested. Therefore, publication bias tests for funnel plot asymmetry are recommended to reach a valid conclusion about publication bias (Begg and Mazumdar, 1994; Egger et al., 1997). Here, there is no publication bias if the *p*-value is not significant at the alpha level (e.g., 0.05). The aim of Rosenthal’s (1979) fail-safe N method is to show the number of unpublished studies required to make the average ES value found statistically significant in the meta-analysis study not statistically significant (Rothstein et al., 2006). The fact that this number, found using the 5*k* + 10 (*k* = sample size), is large indicates that there is no publication bias. Like other statistical analyses, publication bias methods were tested using JASP software version 0.18.3.

## 4 Results

### 4.1 The effects of mindfulness interventions on foreign language achievement

#### 4.1.1 The assessment of publication bias

**Figure 2:**
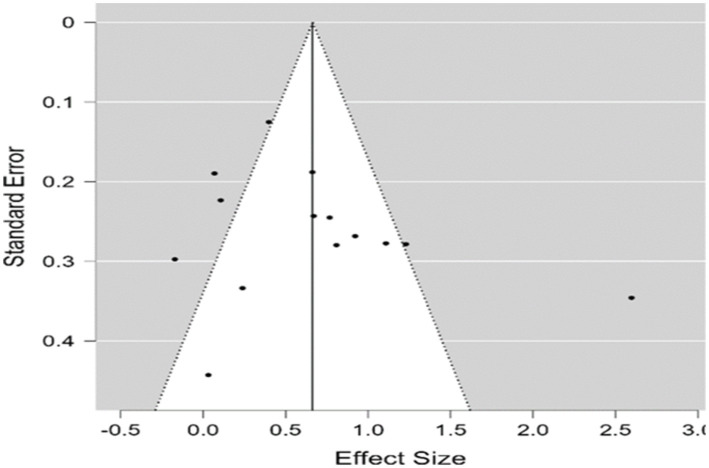
Funnel plot of SE by Hedges’ *g* for intervention studies.

Publication bias was assessed by conducting a funnel plot, Egger’s test, and a rank correlation test, respectively. A funnel plot visually presenting the relationship between standard error and ESs (Hedges’ *g*) in the studies is shown in Figure 2. Publication bias was expected because the relationship was not symmetrically distributed. However, the funnel plot method is not an objective method for publication bias assessment; therefore, Egger’s test and rank correlation tests were also conducted. The findings of the Egger’s test (*z* = 0.821, *p* = 0.412) and rank correlation test (Kendall’s tau = 0.275, *p* = 0.193) were not significant; thus, there is no evidence of publication bias. Moreover, the fail-safe N number was calculated as 463, which is greater than 80 (5 × 14 + 10). This outcome indicates a lack of publication bias as well. It was concluded that publication bias was not an issue in the present study.

#### 4.1.2 Demographic and publication characteristics

**Figure 3:**
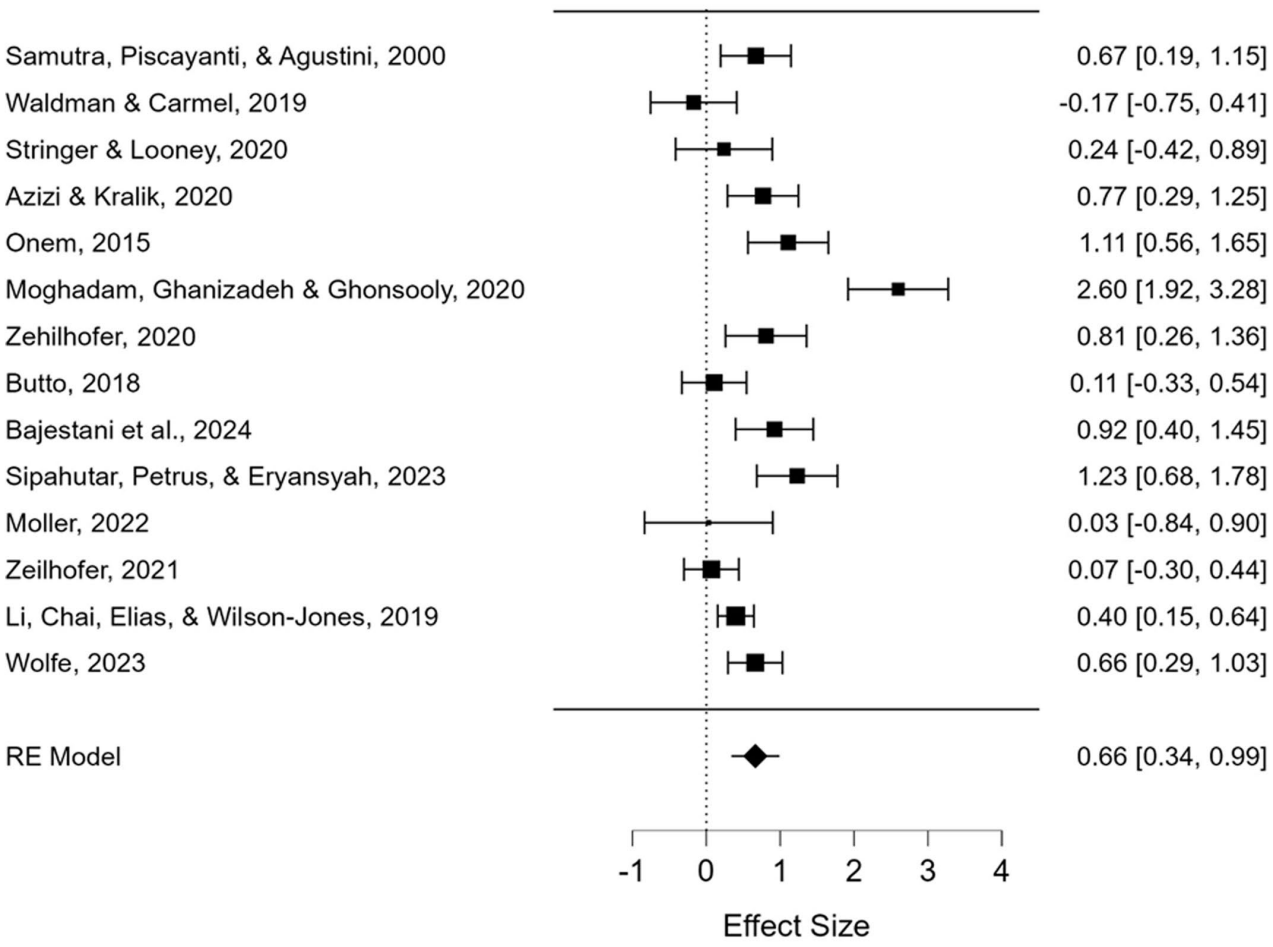
Meta-analysis data and forest plot for interventions.

A total of 14 ESs from 14 studies published in the dataset were calculated in this study. The summary of the ESs of the studies is shown as a forest plot in Figure 3. The study names with authors’ names, the publication year, and ES values by Hedges’ *g* with a 95% confidence interval are reported in the forest plot figure. The findings show that the ES values (Hedges’ *g* values) ranged from −0.017 to 2.60. Applying the random-effects model, the mean ES value was calculated as 0.67 with a 95% confidence interval of 0.34–0.99, which is statistically significant (*z* = 3.971, *p* < 0.001). To test the heterogeneity of the distribution of the ESs, Q-statistics were conducted, and evidence for heterogeneity was statistically provided (Q(1) = 15.772, and *p* < 0.001). The degree of heterogeneity was quantified by the *I*^2^ statistic, and the *I*^2^ value was found to be 84.99, which pointed out a high level of heterogeneity.

#### 4.1.3 Subgroup analyses

For the intervention studies, the effect of region on the mean ES was investigated using the ANOVA approach used for categorical moderators (see Tables 1, 2). The intervention studies in the dataset were conducted in eight countries: Indonesia (*k* = 2), Iran (*k* = 3), Israel (*k* = 1), Japan (*k* = 4), Norway (*k* = 1), Türkiye (*k* = 1), the United States (*k* = 1), and Uzbekistan (*k* = 1). The variable for the region, which was coded as 1 for Middle Eastern countries (Iran, Israel, and Türkiye) and 2 for Far Eastern countries (Indonesia, Japan), was used as a group factor while ES served as a dependent variable in this study. Therefore, Norway, the United States, and Uzbekistan were not included in the analog to the ANOVA analysis of the region variable.

**Table 1:** Study characteristics.

Study	Year	Pub. Type*	Study Design*	Ctry*	N	Edu. Level*	FL*	Mindfulness Data Source	FL Achievement Variable
Azizi and Kralik (2020)	2020	A	Exp.	Iran	45	Uni.	English	Intervention	-Critical reading
Bajestani et al. (2024)	2024	A	Exp.	Iran	60	Uni.	English	Intervention	-Sustained attention -Working memory
Butto (2018)	2018	T	Exp.	Japan	79	Uni	English	Intervention	Reading comprehension
Moghadam et al. (2022)	2020	A	Exp.	Iran	64	Uni.	English	Intervention	-Receptive skills
Møller (2022)	2022	A	Exp.	Norway	22	Uni.	Spanish	Intervention	-Listening comprehension
Önem (2015)	2015	A	Exp.	Türkiye	61	Uni.	English	Intervention	-Vocabulary comprehension
Saputra et al. (2020)	2020	A	Exp.	Indonesia	71	Uni.	English	Intervention	-Writing competency
Sipahutar et al. (2023)	2023	A	Exp.	Indonesia	60	Coll.	English	Intervention	-English achievement
Stringer and Looney (2020)	2020	A	Exp.	Japan	37	Uni.	English	Intervention	-Learning outcomes -Metacognition
Waldman and Carmel (2019)	2019	A	Exp.	Israel	46	Uni.	English	Intervention	-Self efficacy for teaching writing
Wolfe (2023)	2023	T	Exp.	Uzbekistan	60	Uni.	English	Intervention	-Academic performance
Zeilhofer (2020)	2020	A	Exp.	Japan	75	Uni.	German	Intervention	-Academic achievement
Zeilhofer (2021)	2021	A	Exp.	Japan	132	Uni.	German	Intervention	-Foreign language learning
Li et. al. (2019)	2019	A	Exp.	USA	227	Uni.	Chinese	Intervention	-Foreign language learning
Alsharhani et al. (2023)	2023	A	Corr.	Iran	200	Uni.	English	Scales	-Reading comprehension
Charoensukmongkol (2019)	2019	A	Corr.	Taiwan	333	Uni.	English	Scales	-Communication in English
Gao (2023)	2023	A	Corr.	China	492	Uni.	English	Scales	-Self-perceived competence
Hermana and Suganda (2021)	2021	A	Corr.	Indonesia	68	Uni.	English	Scales	-Academic achievement
Navaie et al. (2018)	2018	A	Corr.	Iran	250	Uni.	English	Scales	-Critical thinking
Sheikhzadeh and Khatami (2017)	2017	A	Corr.	Iran	250	Uni.	English	Scales	-Reading comprehension
Tung (2021)	2021	A	Corr.	Vietnam	200	Uni.	English	Scales	-Vocabulary recall -Foreign language performance
Ulivia et al. (2022)	2022	A	Corr.	Indonesia	170	Uni.	English	Scales	-Academic performance
Zeilhofer and Sasao (2022)	2022	Ar	Corr.	Japan	269	Uni.	German	Scales	-Short-term vocabulary retention
**TOTAL N**					3271				

**Table 2:** Moderator analysis for interventions.

Region	*N*	ES	SE	*z*	*p*	95% Confidence Interval	
						Lower	Upper
Middle Eastern	5	1.03	0.39	2.661	0.008	0.27	1.79
Far Eastern	6	0.50	0.17	2.884	0.004	0.16	0.84
Overall	11	0.74	0.21	3.550	<0.001	0.33	1.15

Q_B_ = 1.697, df = 1, *p* = 0.193.

Table 2 points out that the mean of the ES values for the Middle and Far Eastern countries were 1.03 and 0.50, respectively, which are statistically significant (*p* < 0.05). This means that the effectiveness of mindfulness on achievement in foreign language education was significant for the Middle and Far Eastern countries. Between-group Q-statistics were applied to investigate whether ES varies by region. The findings were not statistically significant (Q(1) = 1.697, *p* = 0.193), suggesting that the distribution of ESs was not different between these two subgroups.

### 4.2 The relationship between mindfulness and foreign language achievement

#### 4.2.1 The assessment of publication bias

**Figure 4:**
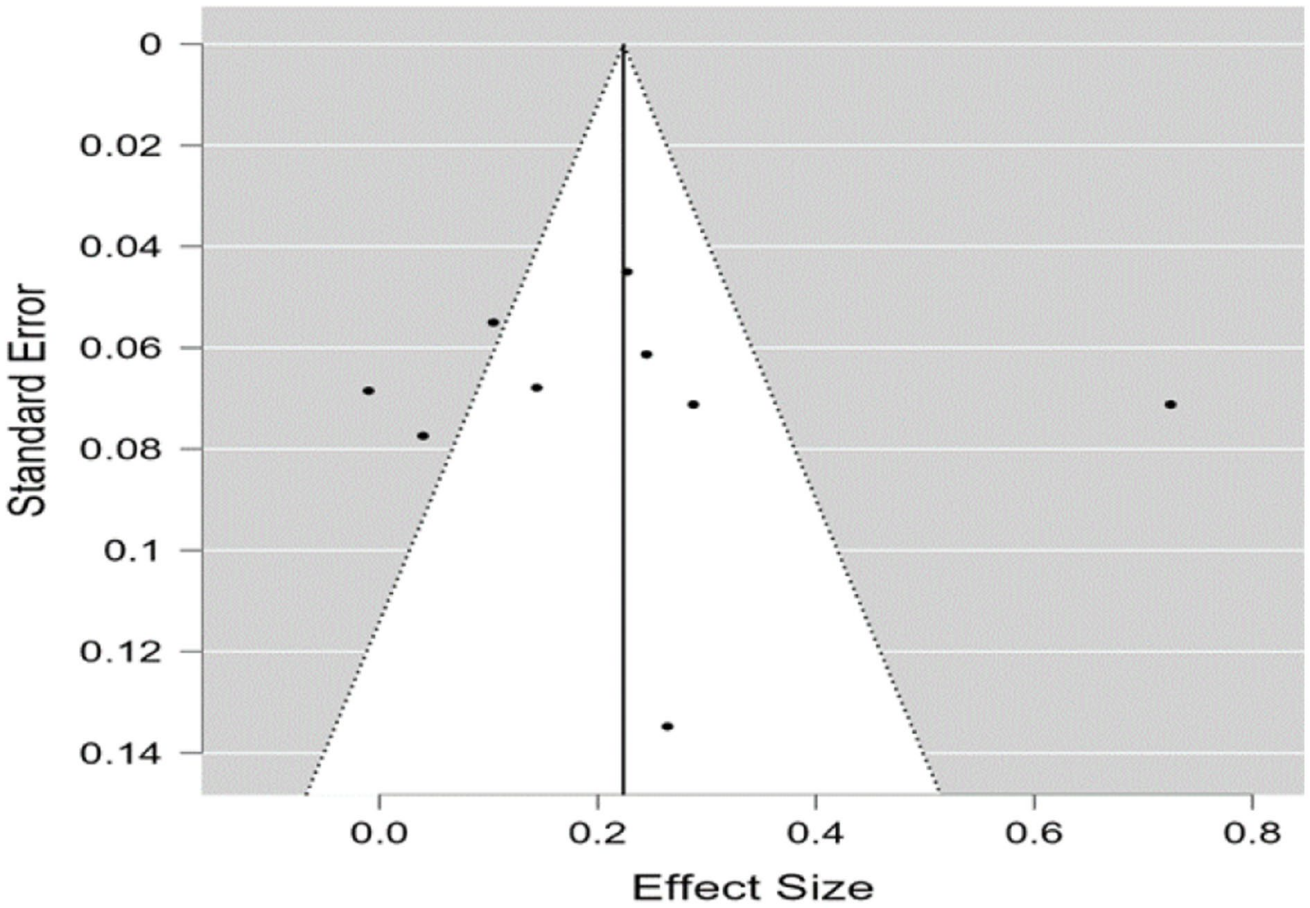
Funnel plot of SE by Fisher’s *z* for correlational studies.

A similar assessment was made for further analysis using a funnel plot, Egger’s test, and a rank correlation test. The funnel plot showing the relationship between the standard error and the ESs (Fisher’s *z*) is shown in Figure 4. Again, the asymmetry suggested a potential risk of publication bias. However, given the subjective nature of funnel plots, further statistical tests were performed. Egger’s test (*z* = 0.263, *p* = 0.792) and the rank correlation test (Kendall’s tau = 0.085, *p* = 0.753) yielded non-significant results, indicating no substantial evidence of publication bias. In addition, the fail-safe N was 315, exceeding the threshold of 55 (5 × 9 + 10), confirming that publication bias was not a major concern in this analysis. Overall, these results indicate that publication bias was not a significant issue in the present study.

#### 4.2.2 Demographic and publication characteristics

**Figure 5:**
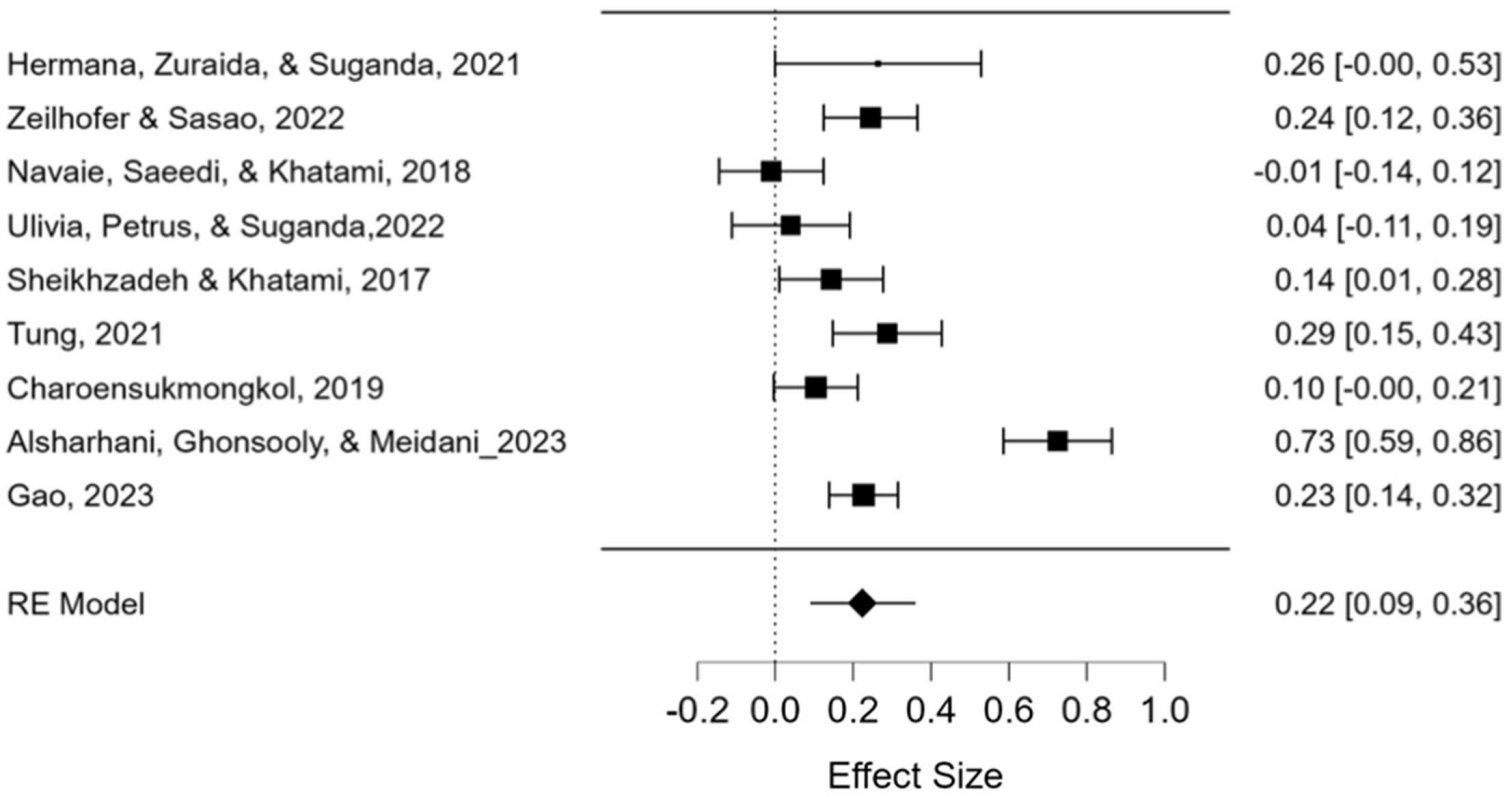
Meta-analysis data and forest plot for correlations.

A total of nine ESs from nine studies were calculated in this study. The summary of the ESs of the studies is shown as a forest plot in Figure 5. The study names with authors’ names, the publication year, and ES values by Hedges’ *g* with a 95% confidence interval are reported in the forest plot. The findings show that the ES values (Fisher’s *z* values) ranged from −0.01 to 0.73. Applying the random-effects model, the mean ES value was calculated as 0.22 with a 95% confidence interval of 0.09–0.36, which is statistically significant (*z* = 3.293, *p* < 0.001). To test the heterogeneity of the distribution of ESs, Q-statistics were conducted, and evidence for heterogeneity was statistically provided (Q(1) = 10.847, *p* < 0.001). The degree of heterogeneity was 89.26, which pointed out a higher level of heterogeneity.

#### 4.2.3 Subgroup analyses

For the correlational studies, the effect of region on the mean ES was investigated using the ANOVA approach used for categorical moderators (see Table 3). The studies were carried out in six countries: China (*k* = 1), Indonesia (*k* = 2), Iran (*k* = 3), Japan (*k* = 1), Taiwan (*k* = 1), and Vietnam (*k* = 1). The variable for the region, which was coded as 1 for Middle Eastern countries (Iran) and 2 for Far Eastern countries (China, Indonesia, Japan, Taiwan, and Vietnam), was used as a group factor while the ES served as the dependent variable in this study.

**Table 3:** Moderator analysis for correlations.

Region	*N*	ES	SE	*z*	*p*	95% Confidence Interval	
						Lower	Upper
Middle Eastern	3	0.29	0.18	1.566	0.117	−0.07	0.64
Far Eastern	6	0.19	0.03	5.523	<0.001	0.12	0.26
Overall	9	0.22	0.07	3.293	<0.001	0.09	0.36

Q_B_ = 0.438, df = 1, *p* = 0.558.

Table 3 shows the mean ES values for Middle and Far Eastern countries were 0.29 and 0.19, respectively. The mean of the ES values for the Middle Eastern countries was not statistically significant (*p* = 0.117), while the mean of the ES values for the Far Eastern countries was statistically significant (*p* < 0.001). That is, the effectiveness of mindfulness on achievement in foreign language education was not significant for the Middle Eastern region but was significant for the Far Eastern region. Between-group Q-statistics were applied to investigate whether ES varies by region. The findings were not statistically significant (Q(1) = 0.438, *p* = 0.558), suggesting that the distribution of ESs was not different between these two subgroups.

## 5 Discussion

The connection between mindfulness and foreign language education and the potential benefits of their collaboration appear promising. However, there is a need for more in-depth investigations to achieve a clear scientific understanding. Existing literature reviews on the role of mindfulness, both with and without interventions, yielded positive findings (Zenner et al., 2014; Koçali and Asik, 2022; Cheng, 2023; Tzelepi et al., 2023), particularly regarding academic performance, language performance, resilience, and foreign language anxiety. Additionally, mindfulness interventions have the potential to enhance all facets of learning, including its cognitive, meta-cognitive, motivational, and emotional dimensions (Moghadam et al., 2022).

The current study explores the significance of mindfulness in foreign language achievement by analyzing and synthesizing the outcomes of existing research to uncover a statistical effect. The overall results yielded a moderate ES of mindfulness on various aspects of foreign language achievement. In the analysis pertaining to R.Q. 1, the meta-analysis demonstrated a statistically significant and positive effect of the interventions on language achievement (*n* = 1,158; Hedge’s *g* = 0.67; 95% CI = [0.34, 0.99]). This implies that integrating mindfulness techniques into language instruction can lead to meaningful improvements in learner performance, and implementing targeted interventions can lead to meaningful gains in students’ language skills. From a practical perspective, this highlights the importance of carefully designing and implementing evidence-based language programs in educational settings. Similarly, for R.Q. 3, the meta-analysis revealed a statistically significant and positive relationship between mindfulness scores and academic achievement (*n* = 2,162; *r* = 0.22; 95% CI = [0.09, 0.35]). Although the evidence is only moderate, it suggests that fostering mindfulness in students may enhance academic performance. Examples of this might be mindfulness exercises in class or teaching techniques. Hedge’s *g* and *r* values also show a positive impact of this approach. After testing how similar the results of ESs for intervention and correlation studies are, *I^2^* was found to be 84.99 and 89.26. This high level of heterogeneity shows that the results are variable in terms of intervention effects and the relationship between mindfulness and academic achievement across the included studies, which may reflect differences in participant characteristics, implementation, measures used, or the specific mindfulness practices examined. Instead of considering an average effect size as applicable universally, it is essential to acknowledge that the effectiveness of mindfulness interventions may vary depending on contextual and methodological differences. So, while the overall effect is important and positive, we need to be careful about how we generalize these findings.

The moderator analysis, conducted to address R.Q.s 2 and 4, examined whether study design (experimental vs. correlational) and geographical region (Middle Eastern vs. Far Eastern) influenced the relationship between mindfulness and foreign language performance. The results show a complex picture. For studies with an experimental design, while both Middle Eastern and Far Eastern countries had statistically significant mean effect sizes, the analysis did not reveal a significant difference in the distribution of effect sizes between the two regions. This suggests that while mindfulness-based interventions may be effective in both regions, the factors influencing the magnitude of these effects may differ or be masked by other variables, such as intervention fidelity and teacher training. In contrast, the correlational studies showed a different pattern. The mean effect size for Middle Eastern countries was not statistically significant, whereas the mean effect size for Far Eastern countries was significant, implying that the association between mindfulness and foreign language achievement was stronger in the Far Eastern region. However, consistent with the experimental findings, the between-group Q-statistic indicated that the overall distribution of effect sizes did not significantly differ between the two regions. This seemingly contradictory finding highlights the potential influence of cultural or contextual factors on the *strength* of the relationship between mindfulness and foreign language learning rather than a fundamental difference in the relationship itself. Particularly, cultural factors toward mental wellbeing and stress management could influence the adoption and effectiveness of mindfulness. In this context, mindfulness and meditation practices have deep historical roots and are more readily accepted and integrated into daily life, potentially leading to greater student engagement and benefit in some Far Eastern cultures. Conversely, in some Middle Eastern contexts, there might be cultural stigma associated with mental health interventions or a lack of awareness and understanding of the benefits of mindfulness, hindering its effective implementation in educational settings. These results also reveal some potential research objectives for mindfulness studies in Western countries where multilingualism is promoted in Europe, particularly in terms of examining the cultural and contextual factors that may influence the effectiveness of mindfulness interventions on language learning.

To some extent, these results are consistent with a similar meta-analytic study by Verhaeghen (2023), which focused on the impact of mindfulness on academic performance through intervention and correlation-based studies and reported average ESs for interventions and significant correlations in the correlational studies. The study results address another meta-analysis of school-based mindfulness interventions (Zenner et al., 2014) that found positive effects, with an overall effect size in which cognitive performance showed the strongest effect, followed by stress reduction and resilience. Zenner et al. (2014) concluded that there is significant heterogeneity, and underpowered studies highlight the need for further research, similar to the present study. In another meta-analysis, Ostermann et al. (2022) also observed a significant effect size for mindfulness interventions on academic achievement. While mindfulness interventions show promise, methodological challenges need to be addressed. By comparison, in a review research on intervention studies on foreign language anxiety, Toyama and Yamazaki (2021) reported that although contemplative practices and relaxation techniques such as mindfulness have been linked to reduced frustration and increased confidence, their role in L2 education remains unclear and further research is needed to confirm their statistical effectiveness.

As the present study scrutinizes the link between mindfulness and foreign language achievement, it contributes to the literature by providing a statistical interpretation by gathering and synthesizing the findings of existing research on the topic. The strength of this research is that it combines studies with different designs and considers bias and heterogeneity. The study advances the theoretical understanding of mindfulness in foreign language teaching by synthesizing empirical research and demonstrating its cognitive, meta-cognitive, motivational, and affective benefits (Moghadam et al., 2022). It extends previous findings on the role of affective and cognitive factors in language learning and is consistent with broader educational psychology research on mindfulness-based learning (Zenner et al., 2014; Verhaeghen, 2023). Practically, the findings highlight mindfulness as an effective pedagogical tool for improving language performance and reducing foreign language anxiety (Toyama and Yamazaki, 2021). Mindful language learning (Zeilhofer and Sasao, 2022) helps people learn better because it helps them feel more relaxed and positive. This fits with Krashen’s affective filter hypothesis, which states that by fostering relaxation, self-confidence, and positive emotions, mindfulness helps create an optimal environment for language acquisition. In addition, the study’s findings suggest that mindfulness is beneficial in different educational contexts, reinforcing its global applicability in language learning and teacher training programs. However, limitations due to the number of included studies suggest an area for improvement in future research.

## 6 Conclusion

As a human-centered perspective of positive psychology, the concept of mindfulness is seen as an assisting method for human mechanisms like learning. In terms of education, mindfulness can serve the human-centered education paradigm that manifests as respect for the person as a whole and nurtures students’ personal qualities and dispositions, such as their inner integrity and harmony (Gill and Thomson, 2017). Considering the relationship between positive psychology and language learning (Wang et al., 2021) and humanistic movement in language teaching (Mercer and MacIntyre, 2014), the significance of the mindfulness approach in foreign language education is a prominent research focus for further studies. Integrating mindfulness techniques into the classroom has profound benefits for students and teachers, fostering an environment of calm, focus, and emotional growth (Takiguchi, 2019). Mindfulness-based contemplative practices have been reported to have promising effects on language success and reduced language anxiety (Scida and Jones, 2017), as they help learners develop greater self-awareness, regulate emotions, and maintain sustained attention. In addition, these practices support language teachers by increasing their own emotional resilience, reducing stress, and improving classroom management, ultimately fostering a more positive and supportive learning environment. Language teachers and curriculum developers can integrate mindfulness techniques, such as focused breathing, meditation, and reflective practices, into teaching strategies to improve learners’ cognitive readiness and emotional resilience. Mindfulness techniques such as focused breathing, meditation, and body awareness exercises have been linked to improved working memory, increased motivation, and enhanced cognitive flexibility, all of which contribute to more effective language learning (MacIntyre and Gregersen, 2012). Meditative practices, such as count-to-ten and guided meditation suggested by Zeilhofer (2020), have shown significant benefits in academic achievement and increased awareness, so language teachers might consider mastering these techniques themselves to serve as practitioners and facilitators, providing students with an additional pedagogical tool for enhanced learning. Therefore, incorporating mindfulness into the language classroom not only supports students’ psychological wellbeing but also empowers teachers, optimizing their ability to facilitate language acquisition with confidence, clarity, and ease.

While previous research and the present study emphasize the numerous advantages of mindfulness, research on its implementation has been argued due to the limitations of the positivist approach in researching mindfulness through interventions; thus, more qualitative and in-depth inquiries are needed (Crawford et al., 2021). For instance, skill-specific interventions such as mindful listening (actively focusing on pronunciation and listening) would be experimental research alternatives for the next attempts. The small number of available studies restricted the investigation of the effects of key variables, highlighting the need for more rigorous primary research in the field, a limitation also noted by Verhaeghen (2023). By handling the limited number of studies with different study designs in the literature, the present study captures mindfulness-based research practices for foreign language achievement to see the overall picture from a statistical perspective.

Future research could consider examining the four language learning skills (speaking, reading, writing, and listening) separately and the affective factors (e.g., anxiety and motivation) that influence foreign language learning from a mindfulness perspective. Further research should consider mindfulness through the lens of positive psychology and humanistic language teaching. Moreover, prospective studies could explore the relationship between mindfulness and foreign language learning in different regions of the world, such as Europe and the US, examining how mindfulness practices affect language learning and whether culturally specific approaches to mindfulness influence foreign language learning differently in different regions.

## Data Availability

The raw data supporting the conclusions of this article will be made available by the authors, without undue reservation.
